# Granulomatous hepatitis, choroiditis and aortoduodenal fistula complicating intravesical Bacillus Calmette-Guérin therapy: Case report

**DOI:** 10.1186/1471-2334-11-260

**Published:** 2011-09-30

**Authors:** Cindy Q Gao, Rozina Mithani, Jack Leya, Lesley Dawravoo, Arvin Bhatia, John Antoine, Felipe De Alba, Peter A Russo, Claus J Fimmel

**Affiliations:** 1 Division of Gastroenterology, Hepatology and Nutrition, Loyola University Medical Center, 2160 South 1st Avenue, Maywood, IL 60153, USA; 2 Department of Pathology, Loyola University Medical Center, 2160 South 1st Avenue, Maywood, IL 60153, USA; 3 Department of Ophthalmology, Loyola University Medical Center, 2160 South 1st Avenue, Maywood, IL 60153, USA; 4 Gastroenterology Section, Edward Hines, Jr. VA Hospital, 5000 South 5th Avenue, Hines, IL 60141, USA; 5 Pathology Section, Edward Hines, Jr. VA Hospital, 5000 South 5th Avenue, Hines, IL 60141, USA; 6 Ophthalmology Section, Edward Hines, Jr. VA Hospital, 5000 South 5th Avenue, Hines, IL 60141, USA

## Abstract

**Background:**

Intravesical instillation of Bacillus Calmette-Guérin (BCG) is the treatment of choice for superficial bladder carcinoma. Complications of BCG therapy include local infections and disseminated BCG infection with multiple endorgan complications.

**Case Presentation:**

We report a case of disseminated, post-treatment BCG infection that initially presented with granulomatous hepatitis and choroiditis. After successful anti-mycobacterial therapy and resolution of the hepatic and ocular abnormalities, the patient developed an acute upper gastrointestinal hemorrhage from an aortoduodenal fistula that required emergency surgery. The resection specimen revealed multifocal, non-caseating granulomas, indicating mycobacterial involvement.

**Conclusions:**

This case highlights the varied end organ complications of disseminated BCG infection, and the need for vigilance even in immuno-competent patients with a history of intravesical BCG treatment.

## Background

Bacillus Calmette-Guérin (BCG) is a live, attenuated strain of the bovine tuberculosis bacillus, *Mycobacterium bovis*. Immunotherapy of neoplastic disease with BCG was developed in the 1960s and has been used for multiple neoplasms including malignant melanoma and acute lymphoblastic leukemia. In 1976, Morales et al. were the first to use intravesical BCG instillation to treat superficial bladder cancer [1]. Since then, BCG therapy has become the treatment of choice for early stage transitional cell carcinoma of the bladder, with response rates ranging from 60%-94% - higher than any chemotherapeutic agent [2].

Although the virulence of attenuated BCG is low, serious and potentially life-threatening infections can occur even in the immunocompetent host. Adverse effects of BCG immunotherapy develop in 3-5% of patients [3]. They include local complications such as bacterial cystitis, bladder contractures, granulomatous prostatitis, epididymitis, orchitis, and systemic reactions such as fever, malaise, hepatitis, and pneumonitis. Disseminated BCG infections presenting as pneumonitis or granulomatous hepatitis, are rare: in the largest retrospective study reported to date, dissemination occurred in 0.7% of over 2,000 patients [4]. Ocular BCG manifestations include uveitis, endophthalmitis, and rarely choroiditis [5,6]. The mechanism of BCG-related choroiditis is unclear - it may be due to a hypersensitivity response or to direct choroidal seeding by the bacteria. Vascular complications of BCG infection are well-known, and include infection and primary aortoenteric fistualization [7,8].

We present a case of *Mycobacterium bovis *hepatitis and choroiditis occurring in an immunocompetent host more than one year after intravesical BCG treatment for bladder cancer. Following prolonged antimycobacterial therapy, the patient developed an aortoduodenal fistula that required emergency surgery and prompted a repeat course of antibiotics. To our knowledge, this constellation of end-organ complications of systemic, BCG infection has not previously been reported.

## Case Presentation

**Figure 1:**
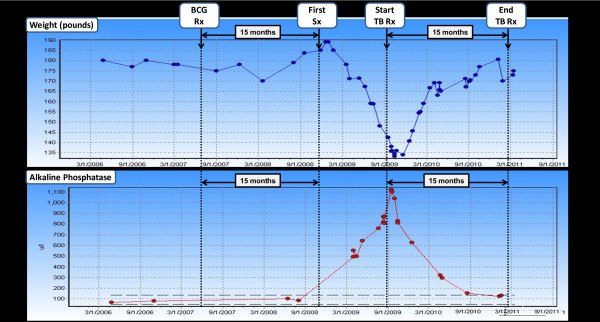
Patient's weight and Alkaline Phosphatase changes prior to BCG instillation, during and after antimycobacterial treatment (BCG Rx: BCG treatment; First Sx: first symptom; TB Rx: TB treatment)

A 61-year-old patient presented to the Hepatology clinic in September of 2008 with a six-month history of elevated alkaline phosphatase levels. He gave a history of malaise, low-grade fever, night sweats and a 50 lbs weight loss (Figure 1). His prior workup included laboratory, radiologic and endoscopic tests, including ultrasound, esophago-gastro-duodenoscopy (EGD) and endoscopic retrograde choledochopancreatography (ERCP), all of which were unrevealing. Serological tests for viral or autoimmune hepatitis, primary biliary cirrhosis, and other concomitant liver diseases were negative.

The patient had no history of immunodeficiencies. He had been diagnosed with superficial transitional cell carcinoma (TCC) of the bladder (Stage T1 with invasion into the lamina propria) in 2007. His initial treatment consisted of cystoscopic fulguration of the tumor. However, follow-up examinations revealed a local recurrence. Intravesical immunotherapy with weekly instillations of BCG (150 mg lyophilized Pasteur strain) was administered for a total of 6 weeks, starting in May, 2007. No immediate complications or traumatic instillations were reported.

On further laboratory testing, the patient's alkaline phosphatase level rose to and peaked at 1100 IU/L (Figure 1). This was associated with mild increase in transaminases (AST 56 IU/L, ALT 60 IU/L). On physical exam, the patient was anicteric. Tender hepatomegaly was present. Aerobic and anaerobic bacterial blood cultures were negative, as was a PCR serum assay for cytomegalovirus. The patient was admitted to the hospital for further workup.

**Figure 2:**
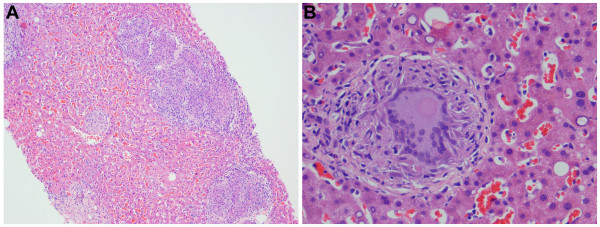
**Pathological features of liver biopsy of granulomatous hepatitis**. Multiple non-caseating granulomas (A, ×10) and typical Langerhans giant cells (B, ×40).

**Figure 3:**
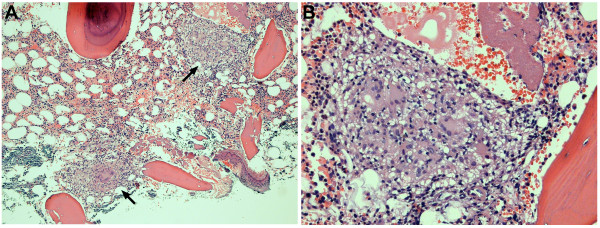
**Bone marrow involvement with *Mycobacterium bovis***. Multiple non-caseating granulomas (arrows) with Langerhans giant cells (A. ×10). B. Higher magnification (×20).

Computed tomography of the chest, abdomen and pelvis was performed to exclude recurrent transitional cell cancer and other malignancies. The lung images revealed multiple small nodules distributed randomly throughout both lungs, suggestive of an infiltrative, possible infectious process. Given the history of prior BCG treatment, the possibility of pulmonary or disseminated mycobacterial infection was raised. A liver biopsy was performed because of the marked liver enzyme elevations, and showed multiple, non-caseating granulomas with Langerhans giant cells (Figure 2); Ziehl-Neelson staining of the liver tissues for acid-fast bacilli (AFB), and Gomori's methamine silver (GMS) staining for fungal organisms with appropriate controls were negative. Because of the continued concern over mycobacterial infection, bone marrow and repeat liver biopsies with mycobacterial culture were performed one month later. Noncaseating granulomas were present on both liver and bone marrow biopsies (Figure 2 and 3). AFB stains were again negative, but mycobacterial cultures were positive in both cases. Mycobacterial isolates from both sources were sensitive to streptomycin, isoniazid, rifampin, and ethambutol. PCR analysis of liver tissue confirmed the presence of *Mycobacterium bovis *BCG DNA, and susceptibility testing demonstrated the expected resistance to pyrazinamide.

**Figure 4:**
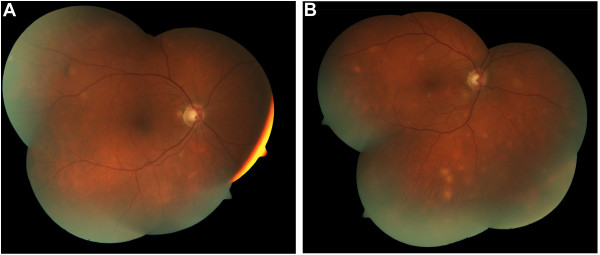
**Ophthalmoscopic findings (multiple, circumscribed yellow-creamy lesions) of bilateral choroiditis with greater severity in the left eye**.

During this stage of his evaluation, the patient reported the new onset of vision changes, consisting of intermittent, painless, blurred vision in both eyes. On ophthalmologic examination, the best corrected visual acuity was 20/20 in the right eye, and 20/30 in the left eye. Vision acuity in the left eye was reduced from pre-existing strabismic amblyopia. Examination of the anterior segment was unremarkable, and intraocular pressures were normal. Fundoscopic examination demonstrated multiple, circumscribed, yellow-creamy lesions of the choroids bilaterally (Figure 4). No vitritis or retinal edema was present. A diagnosis of bilateral, multifocal choroiditis was made and attributed to a rare manifestation of intraocular BCG involvement [6].

Triple anti-mycobacterial therapy was initiated consisting of rifampin (600 mg daily), isoniazid (300 mg daily), and ethambutol (1200 mg daily) for a total of 12 months. On treatment, the patient regained his prior weight, and his physical exam and liver tests normalized (Figure 1). On ophthalmologic examination, the previously noted choroidal lesions had faded. The visual acuity remained unchanged. A follow-up liver biopsy was performed after the completion of anti-mycobacterial therapy. Scattered Langerhans cells were still present, but repeat PCR testing for *Mycobacterium bovis *was now negative.

**Figure 5:**
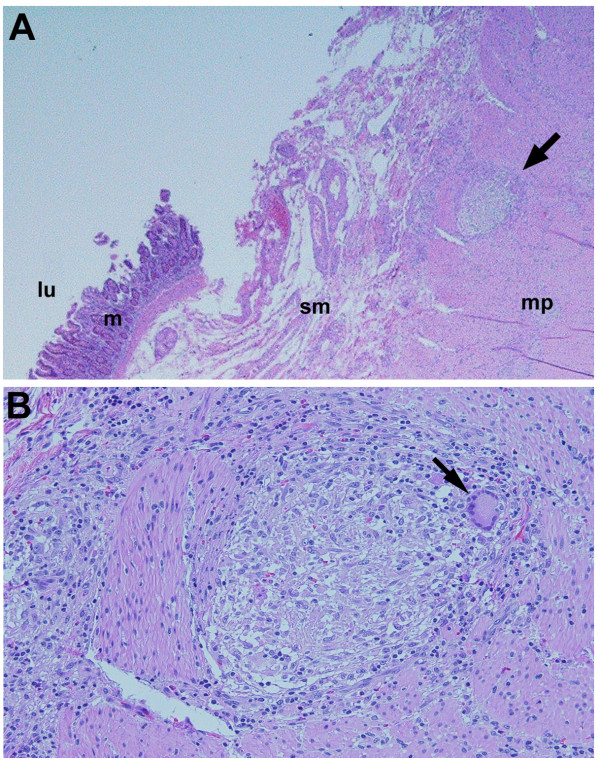
**Duodenal pathology of aorto-duodenal fistula. A.** (×4) single, small, non-caseating granuloma with multinucleated foreign body giant cell reaction (arrow) and chronic transmural inflammation with vascular congestion, consistent with a fistula. L(lumen), m(mucosa), sm(submucosa), mp(musularis propria). **B.** Close-up view of duodenal pathology. (×10) Non caseating granuloma with Langerhans giant cell (arrow).

Two months after the completion of anti-mycobacterial therapy, the patient presented to our hospital with an acute upper gastrointestinal hemorrhage. Emergency endoscopy revealed an adherent clot in the third portion of the duodenum. Abdominal CT examination revealed a previously documented abdominal aortic aneurysm. Compared to a study from one year prior, the diameter of the aneurysm had increased from 2.8 cm in the prior year to 4.1 cm and the aneurysm was impinging upon the third portion of the duodenum (Figure 5). The patient underwent emergency laparotomy, where an aortoduodenal fistualization with fresh clots was encountered. Aortic reconstruction and primary fistula repair were performed successfully. The surgical specimen was sent for pathologic and microbiological studies. Histological examination revealed multifocal, non-caseating granulomas with multinucleated giant cells (Figure 5). Special stains for AFB and fungal organisms were negative. In light of the patient's history of disseminated BCG infection and the presence of multiple granulomas on histological examination, it was felt that the patient warranted retreatment for *Mycobacterium bovis*. He was started on a repeat course of rifampin, isoniazid ethambutol and pyridoxine/B6. The patient is tolerating his treatment well and is currently asymptomatic.

The patient's cultures and stains are all negative but the pathology showed "multifocal, non-caseating small granulomas with multinucleated foreign body giant cell reaction and transmural chronic inflammation with mild vascular congestion, clinically fistula".

## Conclusions

Bacillus Calmette-Guérin (BCG) is an attenuated strain of the tuberculosis bacillus, *Mycobacterium bovis*. BCG induces an intense, localized inflammatory response upon instillation into the bladder. Its therapeutic mechanism of action is thought to involve the ingestion of viable mycobacteria by urothelial cells, which triggers a cytokine-mediated inflammatory response that results in the destruction of tumor cells.

Since its introduction in the 1970s, intravesical BCG treatment has been an effective treatment option of superficial bladder cancer, with a favorable safety profile and typically localized and self-limited side effect [1]. However, systemic complications can occur. They include granulomatous involvement of lung (pneumonititis), liver (hepatitis), and bone marrow [4]. Ophthalmologic and vascular complications have also been described but are considered extremely rare.

According to our literature review, the combination of granulomatous hepatitis, pneumonitis, choroiditis and aorto-enteric fistulization due to disseminated *Mycobacterium bovis *infection has not previously been reported. Our patient was initially referred for a hepatologic evaluation. His liver biopsy demonstrated the typical features of granulomatous hepatitis with numerous epitheloid noncaseating granulomas with intralobar distribution. Despite the negative acid fast stain, a high level of suspicion for mycobacterial infection resulted in further testing and the eventual establishment of multiorgan involvement with *Mycobacterium bovis *BCG. While hepatic, bone marrow, and pulmonary involvement have been extensively documented, the occurrence of mycobacterial choroiditis has only been reported in one prior case report [6]. In this study, a 57 year-old patient developed bilateral ocular lesions that resembled those observed in our patient, following a 6-month course with intravesical BCG with the Connaught strain. Similar to our patient, his choroidal lesions did not progress after therapy with rifampin, isoniazid, and ethambutol [6].

Similarly, the development of an aortoenteric fistula due to disseminated BCG infection is a rare occurrence, with less than 20 cases documented in the literature [8]. The proposed mechanisms of fistulization include hematogenous route via the vasa vasorum or local extension and lymphatic spread [7,9].

Our case highlights the importance of considering unusual complications of BCG therapy and of appropriately monitoring patients at risk.

The proposed mechanisms of liver damage by BCG include direct infection as well as hypersensitivity reactions [10]. In 1996, Leebeek demonstrated the presence of *Mycobacterium bovis *in liver tissues by PCR amplification [11]. This finding supported the concept of hematogenous dissemination and direct tissue damage to the liver [11,12]. However, the beneficial effect of adding corticosteroids to the antituberculous agents and the striking eosinophilic infiltration of the liver suggest that hypersensitivity reactions may also play a role [12,13].^. ^The absence of necrosis in the granulomas has been interpreted as additional evidence of a hypersensitivity reaction. However, hepatic granuloma may resemble those seen in miliary tuberculosis, in which necrosis is usually absent, rather than the caseating lesions of primary tuberculosis with liver involvement. With respect to establishing a diagnosis, our case highlights the limitations of AFB staining and conventional mycobacterial culture. Acid-fast stains are positive in only 10 percent of cases with hepatic involvement, possible due to the fact that a minimum of 10,000 organisms per gram of tissue are necessary to result in a positive stain [12]. Mycobacterial cultures for the detection of BCG lack sensitivity and require a minimum of six to eight weeks of incubation. Molecular assays, including PCR-based tests, have superior sensitivity. However, even they may fail due to the paucity of organisms [12].

With respect to treatment, the recommended first line therapy for severe systemic BCG infection is the administration of isoniazid, rifampin and ethambutol. Treatment with pyrazinamide is not recommended since all known strains of Mycobacterium bovis are pyrazinamide-resistant [10]. Adjunctive therapy with corticosteroids has been advocated by some authors. This recommendation is based on data from animal models and anecdotal clinical reports suggesting that corticosteroids improve the histological outcomes and treatment responses. However, corticosteroid should not be given without concomitant antimycobacterial coverage since they might increase the risk of systemic infection. With respect to prophylactic anti-mycobacterial treatment, the concomitant administration of BCG and antitubercular drugs has been advocated. However, this approach may result in drug-induced hepatitis and reduce the antitumor effects of BCG. Futhermore, systemic BCG infections can occur despite antibiotic prophylaxis.

In summary, we present an unusual case of multi-organ involvement with systemic mycobacterial infection following intravesical BCG treatment of bladder cancer in an immunocompetent patient. The constellation of granulomatous hepatitis, choroiditis and aortoduodenal fistula with gastrointestinal hemorrhage has not been previously reported [3]. Our patient's course highlights the importance of recognizing potential risks from BCG treatment - even months after completion of treatment and in the absence of a history of immunodeficiencies - and of addressing its wide range of end-organ complications.

## Consent

Written informed consent was obtained from the patient for publication of this case report and any accompanying images. A copy of the written consent is available for review by the Editor-in-Chief of this journal.

## Competing interests

The authors declare that they have no competing interests.

## Authors' contributions

CQG and CJF were directly involved in the care and diagnosis of the patient, manuscript preparation, editing and submission. RM contributed to patient care and manuscript preparation. JL, LD, AB were involved in the care of the patient. JA performed the pathologic studies. FA and PAR provided ophthalmologic evaluation and treatment and participated in the preparation of the manuscript. All authors read and approved the manuscript.

## Pre-publication history

The pre-publication history for this paper can be accessed here:

http://www.biomedcentral.com/1471-2334/11/260/prepub
